# Management of Marjolin's ulcer with popliteal lymphadenopathy with surgical resection and lymphadenectomy in a young patient, an uncommon lesion and overlooked entity: A case report

**DOI:** 10.1002/ccr3.7876

**Published:** 2023-09-04

**Authors:** Aroma Naeem, Shehroze Tabassum, Arifa Bibi, Saima Gill, Faiza Afzal, Ayush Anand

**Affiliations:** ^1^ King Edward Medical University Lahore Pakistan; ^2^ Fatima Jinnah Medical University Lahore Lahore Pakistan; ^3^ Mayo Hospital Lahore Pakistan; ^4^ B. P. Koirala Institute of Health Sciences Dharan Nepal

**Keywords:** burns, case report, lymphadenectomy, Marjolin's ulcer, skin neoplasms, surgical resection

## Abstract

**Key Clinical Message:**

In non‐healing ulcers with a previous history of burns, clinicians should have a high index of suspicion for Marjolin's ulcer and a low threshold for biopsy, irrespective of age.

**Abstract:**

Marjolin's ulcer is a rare malignancy arising from chronic inflammation and commonly manifests in burn scars. Thus, in cases of chronic wounds or non‐healing ulcers, health professionals should have a high index of suspicion and a low threshold for biopsy, irrespective of age. Early diagnosis and timely management of tumors can improve the prognosis and overall survival rate. Moreover, further studies are needed to develop an evidence‐based management approach for Marjolin's ulcer.

## Graphical abstract

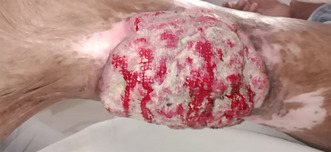

## 1 INTRODUCTION

Marjolin's ulcer (MU) is a cutaneous malignancy resulting from chronic skin inflammation.^1^ A French surgeon, Dr. Jean‐Nicholas Marjolin, was the first to describe the tumor in 1828, leading to its name.^2^ Though the exact incidence of MU in cases of burn scars is unknown, few recent reviews have suggested an incidence of 0.77 to 2%.^3^, ^4^ Males are more affected, and most patients present in the fifth decade of life with a latency period of up to 41 years.^1^, ^3^, ^5^ These patients usually present with non‐healing ulcers.^5^ Such lesions may be overlooked or ignored, and years later, they develop the malignant transformation of cells.^1^ Though MU can occur in any body part, the lower extremities are the most commonly affected.^3^, ^5^, ^6^, ^7^ The mainstay of diagnosing MU is histological analysis via biopsy.^1^, ^3^ MU's most prevalent histological type is squamous cell carcinoma (SCC).^5^, ^6^, ^7^ MU can have a dreadful prognosis, even causing death, as it is often diagnosed in the advanced stage due to its insidious onset.^3^, ^7^ Factors such as tumor grade, tumor site, distant metastasis, and lymph node can determine the prognosis in these patients.^3^, ^7^ The best approach is prevention using early flap surgery or skin grafts in burn cases.^1^, ^3^, ^6^ Despite being a clearly identifiable skin tumor, there remains disagreement on how to treat it.^5^ The preferred treatment approach is wide local excision with a 2–4 cm margin.^8^ If the lymph node is involved, a lymphadenectomy should be done.^9^ Adequate treatment should be accompanied by long‐term follow‐up to detect recurrences.^3^, ^4^, ^9^ Herein, we report the case of Marjolin's ulcer with regional lymphadenopathy in a 22‐year‐old young man following a burn wound scar on the lower extremity.

## 2 CASE REPORT

A 22‐year‐old Asian male presented with a 6‐month history of an enlarging exophytic mass on his right thigh. At the age of 8, he had scald burns involving both his lower extremities, which were treated with debridement and skin grafting and healed completely. About 6 months ago, he suffered trauma to his right thigh, and a raised lesion appeared at the wound site. The lesion increased in size gradually, accompanied by pain and itching. His personal, psychosocial, and drug histories were unremarkable.

**FIGURE 1:**
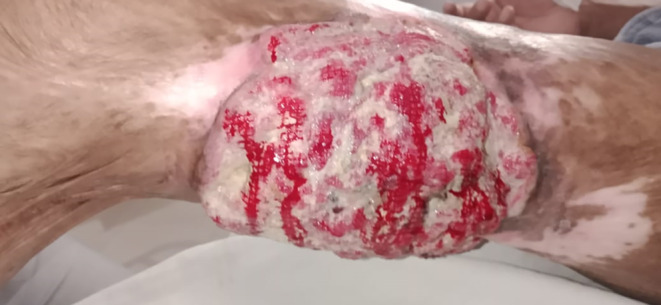
Gross Morphology of Marjolin's ulcer in the patient.

On examination, his vitals were stable. Local examination revealed a 15 × 10 cm circular exophytic mass with bloody discharge on the posteromedial lower one‐third of the right thigh (Figure 1). The distal pulses and sensations were intact. There was no lymphadenopathy, and the rest of the systemic examination was unremarkable.

**FIGURE 2:**
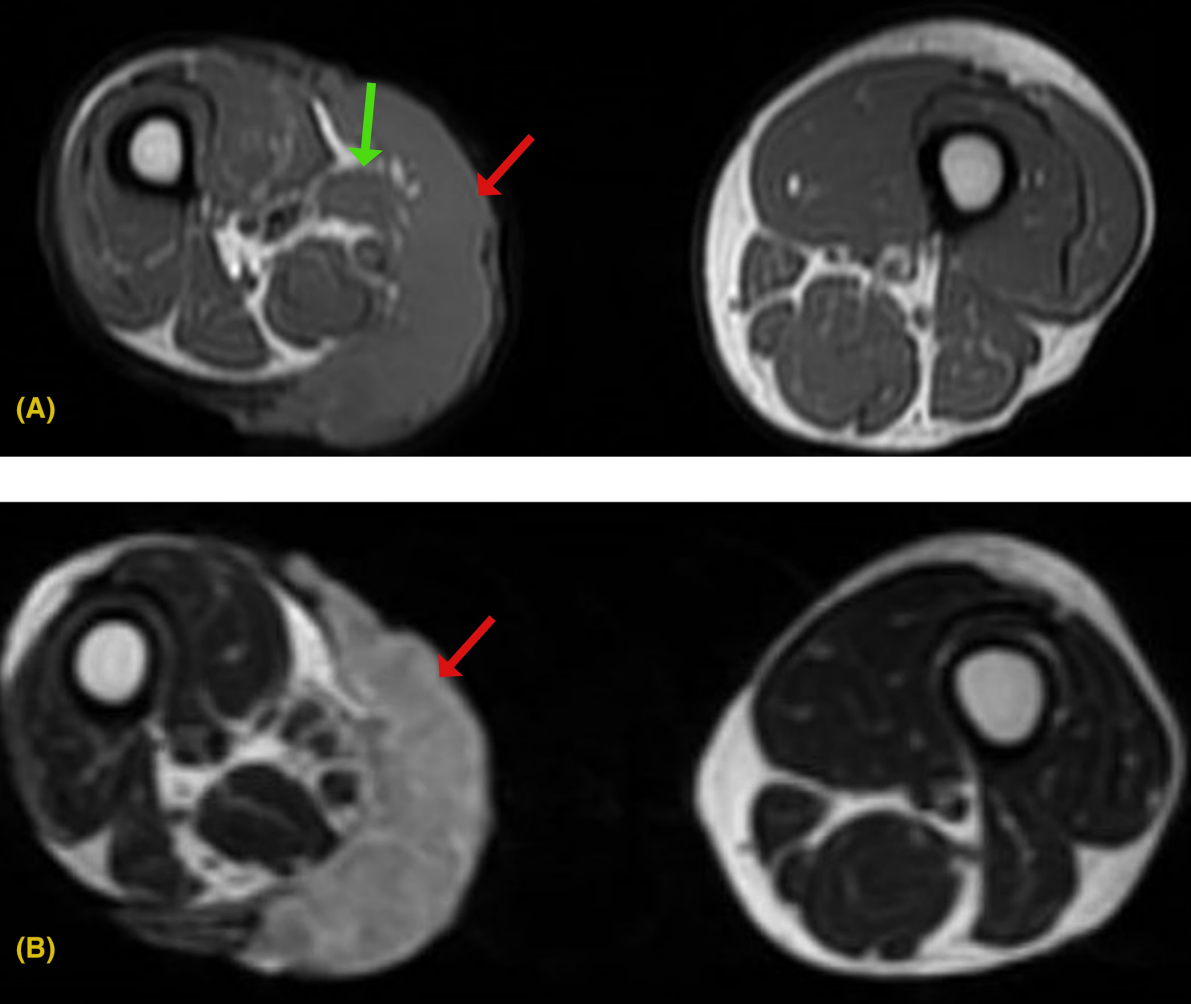
(A) T1W1 axial image showing iso to intermediate intensity mass (red arrow) along the posteromedial aspect of right thigh involving skin, subcutaneous tissue, having loss of fat planes with sartorius (green arrow) and gracilis muscles. (B) T2W1 axial image showing heterogeneously hyperintense mass (red arrow) along the posteromedial aspect of the right thigh involving skin and subcutaneous tissue. Altered signals are also seen in sartorius and gracilis muscles.

**FIGURE 3:**
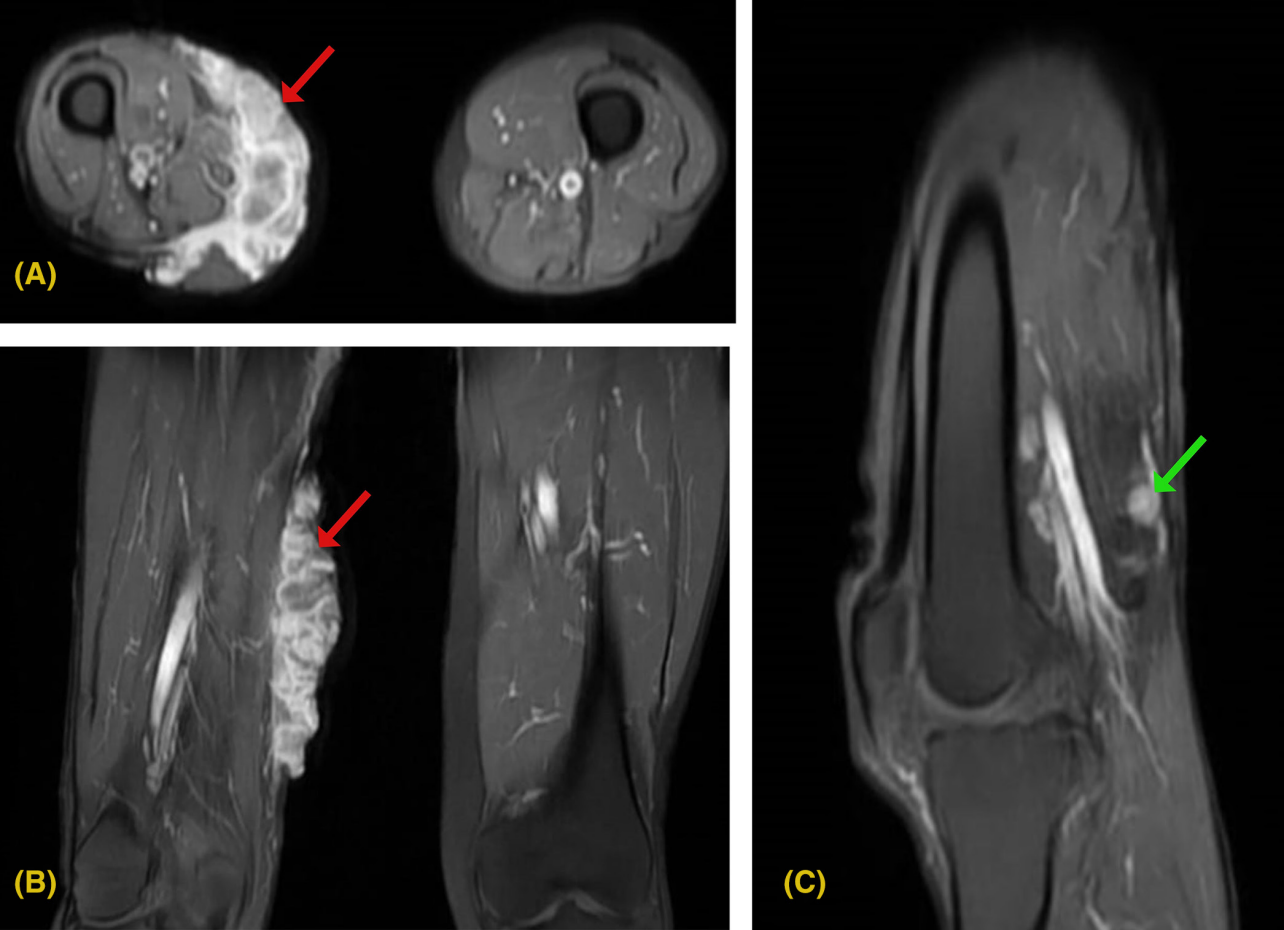
(A,B) T1W1 post contrast axial and coronal sequences showing significant enhancement of right thigh mass which appears lobulated (red arrows). The involved sartorius and gracilis muscles also show enhancement. Popliteal vessels are present deep and are unremarkable. Bone marrow also shows normal signals. (C) Post contrast sagittal view showing enlarged popliteal nodes.

His laboratory investigation showed a total leukocyte count of 14,000 × 10^9^/L and a hemoglobin level of 13 g/dL. We did an incisional biopsy on the mass's anterior, inferior, posterior, and superior edges. Histopathological examination showed groups of squamoid cells with large hyperchromatic nuclei, prominent nucleoli, moderate mitotic activity, and various areas of keratin formation, suggestive of well‐differentiated keratinizing squamous cell carcinoma. To understand the extent of involvement, we did magnetic resonance imaging (MRI) of the right thigh which revealed a mass with an irregular margin in the right thigh (Figure 2) with popliteal lymph node involvement (Figure 3). To evaluate for distant metastasis, we did a computed tomography (CT) scan of the chest, abdomen, and pelvis, which did not reveal distant metastasis.

**FIGURE 4:**
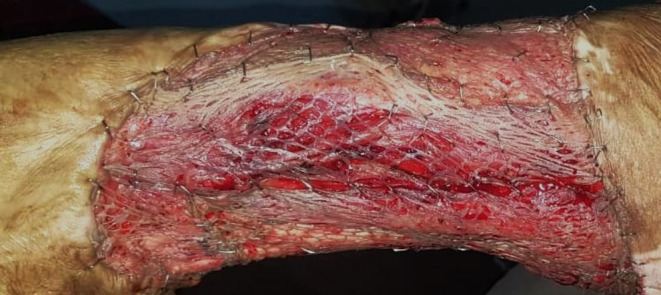
Split thickness skin grafting done in the patient.

After consulting an orthopedician and oncologist, we did a wide local excision of the mass with a 2 cm margin. Since primary closure was not feasible, we did wound closure with a split‐thickness skin graft (Figure 4) taken from the contralateral thigh. Post‐operatively, the patient was kept on radiation therapy for 6 weeks. Following early rehabilitation, the patient was counseled for long‐term follow‐up visits. The patient was lost to follow‐up after 2 months post‐radiotherapy.

## 3 DISCUSSION

The majority of patients with MU present with rapidly growing exophytic non‐healing ulcers.^2^, ^3^, ^5^ In approximately three‐fifths of MU cases, lower extremities are involved.^5^, ^7^ Other sites such as the scalp, face, upper extremities, and trunk can also be involved.^5^, ^7^ The mean age of diagnosis is 51 years (ranging from 30 to 76 years), and most studies suggest a male preponderance.^3^, ^5^ Also, the mean latency period is 22 years (ranging from 11 to 41 years).^5^ The classical finding on local examination reveals everted edge, exophytic growth, and bleeding from the site of the ulcer.^1^, ^2^ However, patients may have an atypical presentation with an irregular base, excess granulation tissues, and increasing lesion size.^1^ The median ulcer size of MU can be 16 × 14 cm.^5^ Our patient was a male who presented with rapidly growing exophytic growth in the lower limb. The presentation was at an earlier age, and the latency period was lower than the mean value reported in the literature.^10^ Local examination revealed classical features of exophytic growth, everted edge, and bleeding, comparable to the median size of the lesion reported in the literature. In addition, careful physical examination to assess lymphadenopathy should be done, as approximately one‐third of patients have a clinically palpable lymph node.^5^ Also, patients can present with distant metastasis involving the chest, abdomen, and brain.^5^ Hence, signs and symptoms suggestive of distant metastases should be considered while evaluating these patients.

A systematic review by Abdi et al. revealed that approximately two‐thirds of patients with MU have a history of burn injury.^5^ MU is approximately reported in 0.7%–2% of burn scars, which suggests the rarity of our case presentation.^3^, ^4^ Various theories have been postulated to explain the development of MU in burn cases. Burn scars may lead to chronic inflammation and compromised blood supply leading to recurrent ulcers and poor healing, ultimately, malignant transformation.^3^ Furthermore, trauma to wounds may also contribute to the development of MU.^3^ Similar to this, our patient had a history of burn injury 14 years back and a history of trauma 6 months ago on the ulcer site. These factors might have contributed to the development of MU in the patient.

Though clinical presentation and examination are essential for initial evaluation, histological analysis via biopsy is the mainstay for diagnosing MU.^1^, ^3^ Histology reveals a well‐differentiated squamous cell carcinoma in most cases.^5^, ^7^, ^11^ Other less commonly reported histological variants are basal cell carcinoma, melanoma, and sarcoma.^11^ In our patient, the histopathology revealed a well‐differentiated SCC. Despite MU being easily diagnosable, various factors such as pre‐existing comorbidities, lack of clinical expertise, and incorrect histology sampling can lead to delays in diagnosis.^12^ In addition to biopsy, imaging modalities such as MRI of the involved site and CT scans of the chest, abdomen, and brain should be used to evaluate the extent of involvement and distant metastasis.^3^, ^5^ We did an MRI of the right thigh, which revealed regional lymphadenopathy and a mass with an irregular margin.

SCC type of MU without metastasis has shown a good prognosis with an overall 3‐year survival rate of 65% to 75% after diagnosis.^13^ However, in the case of metastasis, the survival rate decreases from 35% to 50%.^14^ A recent review showed that death is reported in approximately one in five patients of MU in post‐burn scars.^15^ Hence, early diagnosis and intervention are crucial in these cases. Ideally, prevention is the best approach to avoid the chances of the development of MU. Hence, burn wounds should be managed with skin grafting and flap coverage.^3^

Following the diagnosis of MU, consultation with oncology, dermatology, and plastic surgery should be sought for effective management. To date, no clear guideline states the management approach for MU. Despite no clear consensus, wide local excision with a 2–4 cm margin is the main modality for treatment.^3^, ^5^ In cases where primary closure is not feasible, a skin graft or flap can be used.^5^ In addition, postoperative radiotherapy and rarely chemotherapy have been used.^5^ If there is lymph node involvement, a lymph node dissection should be done.^3^, ^9^ In our case, we managed with wide local excision with a 2 cm margin with lymphadenectomy and wound closure using skin graft followed by postoperative radiotherapy. Despite adequate management, there can be a risk of recurrence in MU. A meta‐analysis showed an increased chance of recurrence in the upper limb, head, and neck tumors.^7^ Also, tumors larger than 10 cm, those with lymph node involvement, and poorly differentiated lesions had increased chances of recurrence.^7^ Our patient had well differentiated large tumor on the lower limb with an enlarged popliteal lymph node. To assess recurrence, long‐term follow‐up is required. We did follow‐up till 2 months post‐radiotherapy, after which the patient was lost to follow‐up.

## 4 CONCLUSION

Marjolin's ulcer refers to the rare malignancy that arises due to chronic inflammation. It commonly manifests in burn scars, with extremities being the most frequently involved. Thus, in cases of chronic wounds or non‐healing ulcers, health professionals should have a high index of suspicion and a low threshold for biopsy, irrespective of age. This can lead to early diagnosis and timely management of tumors, thus improving the prognosis and overall survival rate. There is still a difference of opinion regarding the objective diagnostic criteria and treatment guidelines for Marjolin's ulcer. Hence, there is a need for multicenter clinical collaborative research in this area to make a common evidence‐based consensus.

## AUTHOR CONTRIBUTIONS

**Aroma Naeem:** Conceptualization; data curation; project administration; writing – original draft; writing – review and editing. **Shehroze Tabassum:** Conceptualization; data curation; project administration; writing – original draft; writing – review and editing. **Arifa Bibi:** Conceptualization; data curation; project administration; writing – original draft; writing – review and editing. **Saima Gill:** Conceptualization; data curation; project administration; writing – original draft; writing – review and editing. **Faiza Afzal:** Conceptualization; data curation; project administration; supervision; validation; visualization; writing – review and editing. **Ayush Anand:** Conceptualization; project administration; supervision; validation; visualization; writing – original draft; writing – review and editing.

## CONFLICT OF INTEREST STATEMENT

The authors have no conflict of interest to declare.

## CONSENT

A written informed consent was obtained from the patient based on the journal's policies.

## GUARANTOR OF THE ARTICLE

Faiza Afzal is the guarantor.

## Data Availability

All relevant data pertaining to this case is made available within the manuscript.
